# What Is the Current State of Awareness, Knowledge, and Attitudes Toward Breast Cancer? A Cross-Sectional Survey Among Health and Non-health College Students

**DOI:** 10.3389/fpubh.2022.838579

**Published:** 2022-05-06

**Authors:** Md. Ashraful Islam, Dhfer Mahdi AlShayban, Zeb-un- Nisa, Ghadeer Abdulwahab Mohammed Al-Hawaj, Ghadeer Hani Ali Al-Eid, Atheer Mohammed Moqbel Alenazi, Zubda Malik, Muhammad Bilal Maqsood, Azfar Athar Ishaqui, Zahida Akbar, Muhammad Shahid Iqbal, Mohammad Akbar Hossain, Mohammed Alnuhait, Abdul Haseeb

**Affiliations:** ^1^Department of Pharmacy Practice, College of Clinical Pharmacy, Imam Abdulrahman Bin Faisal University, Dammam, Saudi Arabia; ^2^Faculty of Pharmacy, Ziauddin University, Karachi, Pakistan; ^3^College of Clinical Pharmacy, Imam Abdulrahman Bin Faisal University, Dammam, Saudi Arabia; ^4^Department of Urology, Liaquat National Hospital and Medical College, Karachi, Pakistan; ^5^King Abdullah International Medical Research Center-Eastern Region, Al Ahsa, Saudi Arabia; ^6^King Saud Bin AbdulAziz University for Health Sciences, Ministry of National Guard Health Affairs, Al Ahsa, Saudi Arabia; ^7^Department of Pharmacy, Iqra University, Karachi, Pakistan; ^8^Department of Medicine, Ministry of National Guard Health Affairs, Al Ahsa, Saudi Arabia; ^9^Department of Pharmacy, King Faisal General Hospital, Al Ahsa, Saudi Arabia; ^10^Department of Obstetric and Gyneacology, Ministry of National Guard Health Affiars, Al Ahsa, Saudi Arabia; ^11^Department of Clinical Pharmacy, College of Pharmacy, Prince Sattam Bin Abdulaziz University, Alkharj, Saudi Arabia; ^12^Department of Pharmacology and Toxicology, Al-Qunfudah Medical College, Umm Al Qura University, Al Qunfudah, Saudi Arabia; ^13^Department of Clinical Pharmacy, College of Pharmacy, Umm Al Qura University, Makkah, Saudi Arabia

**Keywords:** breast cancer, health knowledge (source: MeSH, NLM), attitudes, awareness of disease, students, Saudi Arabia (KSA)

## Abstract

**Objective:**

To document breast cancer (BC) knowledge, awareness, and attitudes among female undergraduate students studying at health and non-health colleges.

**Methods:**

A 3-month cross-sectional study was conducted among female undergraduate students studying at health and non-health subject colleges affiliated to a public university. Convenience sampling was employed, and a previously validated questionnaire available in English and Arabic languages was used. Multiple linear regression was used to report the predictors of BC knowledge. A two-tailed *p*-value of < 0.05 was considered significant. The study was approved by an ethics committee.

**Results:**

A total of 506 responses were analyzed. The mean knowledge score was 13.98 ± 4.1. The findings of the surveyed students suggested that more than 55% of the students had an acceptable level of knowledge. By education sector, approximately 70% and 40% of health and non-health college students, respectively, had an acceptable level of knowledge. The mean difference in knowledge scores between students of health and non-health colleges was significant (*p* < 0.001) as students at health colleges had a higher score. Age, college type and the presence of the disease in family/relatives were significant predictors of students' BC knowledge (*p* < 0.05).

**Conclusion:**

By comparing it with previous evidence, the knowledge of BC has improved. The role of awareness campaigns as an information medium for students from non-health backgrounds is greatly appreciated. Moreover, the internet and electronic media have emerged as new sources of information for non-health college students, and therefore, more efforts are needed to utilize this medium in empowering this student population in understanding of this disease.

## Introduction

One of the most common cancers in women worldwide is breast cancer (BC) (1). In addition, it is the leading cause of cancer death in women (1–3). According to 2019 statistics from the Institute for Health Metrics and Evaluation (IHME), deaths due to BC accounted for 2.66% (2.49–2.79%) of all deaths among women of all ages globally, with an annual percentage change of 0.81%. Among women aged 15–49 years, deaths attributable to BC accounted for 4.93% (4.68–5.18%) of all deaths with an annual percentage change of 0.26%. In addition, among women aged 50–69 years, deaths due to BC accounted for 5.25% (5–5.49%) of all deaths, with an annual percentage change of −0.42% at the time of this writing (4).

Evidence has reported that most deaths due to BC occur in developing countries, and that most patients present with BC in the late stages (1). Studies show that low knowledge of this type of cancer and some cultural barriers are the potential reasons for the late presentation of BC among women in developing countries (2, 5). In addition, in many developing countries, the lack of effective screening programs leads to high mortality and delayed diagnosis (6). Berry et al. cited screening mammography and management as the core reasons for the decline in mortality due to BC in the USA from 1975 to 2000 (7). The World Health Organization (WHO) cites early detection as the keystone of increasing chances of better health outcomes and survival in BC (3). Therefore, knowledge of the ailment and awareness of detection techniques are essential for diagnosing BC at an early stage that would increase the chances of survival. However, research among women in Arab countries have highlighted a low knowledge of this disease (5, 8).

Available evidence cited BC as the second leading cause of cancer-related deaths in Saudi Arabia (9). According to 2019 statistics from IHME, deaths attributed to BC were 3.28% (2.76–3.88%) of total deaths among Saudi women of all ages, with an annual percentage change of 2.69%. Meanwhile, the highest percentage was reported among women aged 15–49 years, i.e., 4.35% (3.44–5.41%) BC-related deaths out of total deaths with an annual percentage change of 1.82% (10). A mortality rate of 41.26 (29.61–55.69) per 100,000 deaths due to BC was reported among Saudi women aged 50–69 years (10). All above mentioned figures were captured at the time of this writing.

According to the 2017 Cancer Incidence Report of Saudi Arabia, BC was the leading cause of death in women in 2017, with 2,463 women BC cases reported between January and December 2017. BC accounted for 17.7% of all reported malignancies among Saudis and 30.9% of all reported cancers in women of all ages. The Saudi women population had an age-standardized incidence rate (ASR) of 29.7/100,000. The five regions with the highest figure are the Eastern Region that has the highest ASR per 100,000 people at 52.2/100,000, followed by Riyadh at 37/100,000, Qassim at 34.8/100,000, Makkah at 31.4/100,000, and Al Jouf at 20.8/100,000 (11).

It has been well documented in studies that there is a lack of knowledge of BC among Saudi academy students. Previous studies have reported a low knowledge among Saudi women students (9, 12). However, those studies did not differentiate among health and non-health college students and did not use a validated research instrument to document knowledge. In a study conducted in Najran, it was reported that students and faculty had a better general knowledge of BC, but poor knowledge about its symptoms (13). In addition, there is a plethora of evidence that highlights these issues among the general Saudi public as well. A study in Jeddah reported that the level of knowledge about BC was inadequate (14). Similar findings were obtained in another study on Saudi women hailing from the Qassim region (15). To this end, a study reported that few women opted for free screening offered at hospitals owing to several socioeconomic and health-related factors (16). A systematic review reported several reasons, namely, fear, ignorance, and discomfort in discussing the topic that contributed to this knowledge debacle (17).

This occurrence was established as a scientific fact. The Imam Abdulrahman Bin Faisal University (IAU) routinely conducted BC awareness campaigns (18–20). Since 2015, such campaigns have been a regular occurrence at universities, where notable healthcare professionals and academicians with expertise in this field are invited to disseminate knowledge of the ailment among faculty and students. Hence, it was worthwhile to note that how such campaigns would impact the knowledge of students in this domain. In addition, the shortcomings observed in previous studies, such as no differentiation of health and non-health background and the use of non-validated instruments, were considered in the design of this study.

## Methods

### Study Objective

This study documented BC knowledge, awareness, and attitudes from female undergraduate students studying at health and non-health subject colleges.

### Study Design, Duration, and Venue

A cross-sectional study of a 3-month duration, i.e., September–November 2020, at health and non-health subject colleges affiliated to the IAU.

### Participants

The target participants for this study were female students aged >18 years currently enrolled in health and non-health colleges of the university. Graduates and dropouts were not included. Pregnant students, students with any illnesses, or students who were not willing to participate were not included.

### Research Hypothesis and Objectives

It was hypothesized that because the university routinely conducts awareness campaign pertaining to the BC, the knowledge of the students, including those studying in health and non-health colleges of this university, would be better as compared to previous findings in local and regional contexts. Moreover, it was also hypothesized that students' attitudes toward screening and treatment would be positive.

### Sampling Strategy and Sample Size Calculation

Convenience sampling procedure was used to collect the data. According to official data, a total of 30,062 students were enrolled in 2020 at the time of this writing (21). From this known population, the sample size was calculated and adjusted for potential nonresponse rate using formulae (22) and (23) as follows:


$$\begin{array}{lll} \;n\; & = & \;\frac{N}{1\;+\;Nd^{2}}\;and,\\ n_{n}\; & = & \;\frac{n}{1\;-\;d} \end{array}$$

where *n* = required sample size, *N* = population size, and *d* = margin of error (ideal value is 0.05) for 95% confidence level, *n*_*n*_ = sample size after accounting for the nonresponse rate, *n* = calculated sample size, and *d* = nonresponse rate. The final sample required after the calculation was 493.42 in this study. Initially, we managed to collect responses from 510 samples in this study.

### Research Instrument and Translation

We used a previously developed and validated research instrument known as the “BC Inventory” with permission (2). The questionnaire had three sections, namely, knowledge, awareness, and attitudes. The knowledge section contained 11 multiple-choice questions and was graded. Each item had a score, and the sum of all scores from the 11 items yielded a final knowledge score. The total score corresponded to the respondent's level of knowledge. The maximum achievable score was 24, and a higher score represented a higher level of knowledge. The cut-offs were defined as poor knowledge (0–7), low knowledge (8–13), adequate knowledge (14–18), and excellent knowledge (19–24). We considered poor and low knowledge as an unacceptable level and adequate and excellent knowledge as an acceptable level. Awareness was assessed by the six items that included variable response levels, i.e., 3–6. In addition, 10 items of different response levels documented attitudes among the participants (2, 24).

First, the English version of the questionnaire was rechecked for consistency with the original Urdu version. The English version of the questionnaire was then translated into the Arabic language by three native Arabic speakers who were subject matter experts. This version was verified by reverse translation by two reviewers who were native Arabic speakers and spoke English as a second language. At this point, any inconsistencies have been addressed. The final version was subsequently produced and piloted among a group of students. There was no difficulty in reading and understanding the questionnaire. The questionnaire was deemed fit to use at this stage. The pilot results were not included in the final analysis.

### Data Collection

The data were collected by distributing the questionnaire through a physical survey at the time when students attended an exam or had any academic activity on-campus. At the same time, an online link was also distributed to students. Students had the choice of responding through either a physical or an online survey whichever seemed convenient. The physical survey was returned to the designated person either at the same time or at a time of convenience for the participants. The survey data were gathered and entered into statistical software along with the online survey data at the end of the data collection period.

### Data Management and Statistical Methods

Data were entered into IBM SPSS, version 23 (IBM Corp., Armonk, NY, USA), for analysis and interpretation. Missing and abnormal values in the data were cleaned using the informal technique. Data normality and outliers were tested using both formal, i.e., the Kolmogorov–Smirnov test, and informal, i.e., graphical visualization techniques (25, 26). Frequency distributions were performed on background characteristics, and the results were presented as sample counts (*N*) and percentages (%) while scale data were assessed by calculating the mean (*X*) with SD and/or median with IQR where it was applicable. Multiple linear regression was used to find significant predictors of BC knowledge. Only significant variables were included in the final model from a bivariate *t*-test. The cut-off value of variance inflation factor (VIF) <5 was used to detect multicollinearity among predictors (27). In addition, other model assumptions, such as linearity and homoscedasticity, were checked using scatter plots. A two-tailed *p* < 0.05 was considered significant. Knowledge score was the main outcome variable. The variables of awareness and attitudes were secondary outcome variables.

### Ethics Approval and Consent

The study was approved by the Institutional Review Board of the King Abdullah International Medical Research Center (Study# RA20/020/A) and (Reference# IRBC/1325/20) on August 11, 2020 through an expedited review. All students were provided with an informed consent form at the time of participation. Those who consented to participate were provided with a questionnaire.

## Results

A total of 506 samples were analyzed after data cleaning.

### Background Characteristics

Most students were 20 years and above (*N* = 391, 77.3%), studied at health colleges (*N* = 309, 61.1%), and had no family members with BC (*N* = 397, 78.5%). Details are presented in Table 1.

**Table 1:** Background characteristics of participants (*N* = 506).

**Characteristics**	**Frequency (*N*)**	**Percent (%)**
**Age in years**		
≤ 20 years	115	22.7
> 20 years	391	77.3
**College**		
Health colleges	309	61.1
Non-health colleges	197	38.9
**Marital Status**		
Single	373	73.7
Married	133	26.3
**Study year**		
Preparatory year	35	6.9
2^nd^ year	67	13.2
3^rd^ year	94	18.6
4^th^ year	163	32.2
5^th^ year	118	23.3
6^th^ year	29	5.7
**Monthly Family income**		
SAR <5,000 (USD <1333.33)	43	8.5
SAR ≥ 5,000–10,000 (USD ≥ 1333.33–2666.27)	226	44.7
SAR > 10,000 (USD > 2666.27)	237	46.8
**Any family or relatives having breast cancer?**		
Yes	109	21.5
No	397	78.5
*1 USD equals SAR 3.75*		

### Awareness of BC

About 40% of the students mentioned that they were fully aware of BC (*N* = 202, 39.9%), but more than half were partially aware (*N* = 279, 55.1%). One-third of the students were fully aware of mammography (*N* = 158, 31.2%), but a considerable proportion was partially aware (*N* = 213, 42.1%). Fewer than half were fully aware of BC screening (*N* = 233, 46%), followed by a similar number of partially aware students (*N* = 239, 47.2%). More than half of the students were fully aware of breast self-examination (BSE; *N* = 278, 54.9%). One-third were aware of the treatment (*N* = 174, 34.4%), followed by a considerable number of partially aware students (*N* = 232, 45.8%). The most common source of information for students in this matter was healthcare professionals (*N* = 134, 26.5%). The Internet and electronic media were the most common sources of information for students at non-health colleges (*N* = 66, 33.5%). Data on awareness regarding BC are presented in Table 2.

**Table 2:** Awareness of breast cancer (BC) (*N* = 506).

**Items**	**Frequency (%)**		
	**All sample** **(*n* = 506)**	**Health subjects (*n* = 309)**	**Non-health subjects** **(*n* = 197)**
**Awareness regarding breast cancer**			
Yes, I am aware of it	202 (39.9)	153 (49.5)	49 (24.9)
My awareness is not adequate	279 (55.1)	151 (48.9)	128 (65)
Not aware	25 (4.9)	5 (1.6)	20 (10.2)
**Awareness regarding mammography**			
Yes, I am aware of it	158 (31.2)	124 (40.1)	34 (17.3)
My awareness is not adequate	213 (42.1)	125 (40.5)	88 (44.7)
Not aware	135 (26.7)	60 (19.4)	75 (38.1)
**Awareness regarding screening for breast cancer**			
Yes, I am aware of it	233 (46)	162 (52.4)	71 (36)
My awareness is not adequate	239 (47.2)	137 (44.3)	102 (51.8)
Not aware	34 (6.7)	10 (3.2)	24 (12.2)
**Awareness regarding breast self-examination**			
Yes, I am aware of it	278 (54.9)	191 (61.8)	87 (44.2)
My awareness is not adequate	172 (34)	96 (31.1)	76 (38.6)
Not aware	56 (11.1)	22 (7.1)	34 (17.3)
**Awareness regarding breast cancer treatment**			
Yes, I am aware of it	174 (34.4)	136 (4)	38 (19.3)
My awareness is not adequate	232 (45.8)	134 (43.4)	98 (49.7)
Not aware	100 (19.8)	39 (12.6)	61 (31)
**Source of information regarding breast cancer**			
Friends, acquaintances, colleagues, and coworkers	39 (7.7)	20 (6.5)	19 (9.6)
Newspapers, literature	101 (20)	58 (18.8)	43 (21.8)
Internet and electronic media	125 (24.7)	59 (19.1)	66 (33.5)
Healthcare professionals	134 (26.5)	105 (34)	29 (14.7)
Family and relatives	104 (20.6)	67 (21.7)	37 (18.8)
Other sources	3 (0.6)	0 (0)	3 (1.5)

### Attitudes Toward BC

The most common reason to undergo breast screening was fear (*N* = 138, 27.3%), followed by breast pain (*N* = 77, 15.2%). More than half of the students felt that it was better to have BC screening in a hospital or clinic (*N* = 292, 57.7%). Most students mentioned that they did not feel embarrassed talking about BC (*N* = 473, 93.5%) but did not discuss this issue with their doctor (*N* = 466, 92.1%). Most students preferred to discuss the issue with a female doctor (*N* = 354, 70%). The most common barrier to breast screening was fear of diagnosis (*N* = 130, 25.7%). Most students recommended breast screening to others (*N* = 397, 78.5%). Half of the students acknowledged it as a major public health problem in the country (*N* = 217, 53.6%). Details of the attitudes of students are presented in Table 3.

**Table 3:** Attitudes of students toward BC (*N* = 506).

**Items**	**Frequency (%)**		
	**All sample** **(*n* = 506)**	**Health subjects (*n* = 309)**	**Non-health subjects (*n* = 197)**
**What do you perceive to be the main reason for you to undergo breast screening?**			
Fear of having the disease	138 (27.3)	78 (25.2)	60 (30.5)
Media awareness	41 (8.1)	22 (7.1)	19 (9.6)
Doctor / pharmacist's advice	27 (5.3)	20 (6.5)	7 (3.6)
Breast pain	77 (15.2)	46 (14.9)	31 (15.7)
Advice of friend, husband, etc.	4 (0.8)	0 (0)	4 (2)
Breast cancer in the family	46 (9.1)	28 (9.1)	18 (9.1)
More than one reason	173 (34.2)	115 (37.2)	58 (29.4)
**How do you feel about breast screening at a hospital or clinic?**			
Yes, it is better	292 (57.7)	189 (61.2)	103 (52.3)
Only when the need arises	207 (40.9)	117 (37.9)	90 (45.7)
It is culturally unacceptable	1 (0.2)	0 (0)	1 (0.5)
I have religious issues in doing so	6 (1.2)	3 (1.0)	3 (1.5)
**Do you feel embarrassed talking about breast cancer in the society?**			
Yes	33 (6.5)	17 (5.5)	16 (8.1)
No	473 (93.5)	292 (94.5)	181 (91.9)
**Did you ever discuss this disease with your doctor?**			
Yes	40 (7.9)	29 (9.4)	11 (5.6)
No	466 (92.1)	280 (90.6)	186 (94.4)
**If need be, who would you prefer to discuss it with?**			
Female doctor	354 (70)	198 (64.1)	156 (79.2)
Male doctor	2 (0.4)	1 (0.3)	1 (0.5)
Anyone	150 (29.6)	110 (35.6)	40 (20.3)
**If you ever undergo breast screening what would be your trigger factor (main reason)?**			
Fear of the disease	145 (28.7)	83 (26.9)	62 (31.5)
Assurance of not having breast cancer	225 (44.5)	136 (44.0)	89 (45.2)
Diagnosis after suspicion	109 (21.5)	70 (22.7)	39 (19.8)
More than one reason	27 (5.3)	20 (6.5)	7 (3.5)
**What do you perceive to be barriers to undergoing breast screening?**			
Culture / traditions	121 (23.9)	62 (20.1)	59 (29.9)
Shyness / Reluctance	74 (14.6)	30 (9.7)	44 (22.3)
No facilities	62 (12.3)	49 (15.9)	13 (6.6)
No female doctor/ Not to be examined by a male doctor	33 (6.5)	26 (8.4)	7 (3.6)
Lack of self-knowledge	83 (16.4)	50 (16.2)	33 (16.8)
Conservative society	3 (0.6)	2 (0.6)	1 (0.5)
Fear of diagnosing the disease	130 (25.7)	90 (29.1)	40 (20.3)
**What would be your advice to others regarding the issue?**			
Do not undergo breast screening	78 (15.4)	48 (15.5)	30 (15.2)
Must undergo breast screening	397 (78.5)	245 (79.3)	152 (77.2)
I cannot comment on it	31 (6.1)	16 (5.2)	15 (7.6)
**Do you think it is becoming a major problem in Saudi Arabia?**			
Yes	271 (53.6)	170 (55.0)	101 (51.3)
No	41 (8.1)	23 (7.4)	18 (9.1)
Maybe	194 (38.3)	116 (37.5)	78 (39.6)

### Knowledge of BC

The mean knowledge score was 13.98 ± 4.1, the minimum score was 3, and the maximum score was 23. Details of the knowledge of students are presented in Table 4.

**Table 4:** Knowledge of BC (*N* = 506).

**Items**	**Frequency (%)**			
		**All sample** **(*n* = 506)**	**Health subjects (*n* = 309)**	**Non-health subjects** **(*n* = 197)**
**Ever heard about breast cancer?**				
Yes		503 (99.4)	307 (99.4)	196 (99.5)
No		3 (0.6)	2 (0.6)	1 (0.5)
**Knowledge of screening programs for breast cancer?**				
Yes, I am aware of it		216 (42.7)	142 (46)	74 (37.6)
My awareness is not adequate		232 (45.8)	142 (46)	90 (45.7)
Not aware		58 (11.5)	25 (8)	33 (16.8)
**Heard about breast self-examination (BSE)?**				
Yes, I am aware of it		438 (86.6)	278 (90)	160 (81.2)
My awareness is not adequate		58 (11.5)	29 (9.4)	29 (14.7)
Not aware		10 (2)	2 (0.6)	8 (4.1)
**Ever performed breast self-examination (BSE) at home?**				
Yes		258 (51)	167 (54)	91 (46.2)
No		248 (49)	142 (46)	106 (53.8)
**Knowledge of breast cancer treatments?**				
Yes, I am aware of it		190 (37.5)	144 (46.6)	46 (23.4)
My awareness is not adequate		240 (47.4)	138 (44.7)	102 (51.8)
Not aware		76 (15)	27 (8.7)	49 (24.9)
**Ever underwent breast screening at hospital or clinic?**				
Yes		42 (8.3)	31 (10)	11 (5.6)
No		464 (91.7)	278 (90)	186 (94.4)
**Heard about mammography?**				
Yes		320 (63.2)	224 (72.5)	96 (48.7)
No		186 (36.8)	85 (27.5)	101 (51.3)
**Can breast cancer spread to other parts of the body?**				
Yes, it can spread		362 (71.5)	225 (72.8)	137 (69.5)
No, it cannot spread		58 (11.5)	36 (11.7)	22 (11.2)
I am not sure		86 (17)	48 (15.5)	38 (19.3)
**Does overweight and obesity increase the risk of having breast cancer?**				
Yes		250 (49.4)	171 (55.3)	79 (40.1)
No		256 (50.6)	138 (44.7)	118 (59.9)
**Most common symptom of breast cancer**				
Pain and redness		59 (11.7)	38 (12.3)	21 (10.7)
Change in color and size of the nipple		23 (4.5)	11 (3.6)	12 (6.1)
Change in breast size		139 (27.5)	100 (32.4)	39 (19.8)
I do not know		285 (56.3)	160 (51.8)	125 (63.5)
**Most common risk factor for breast cancer**				
Family history		161 (31.8)	132 (42.7)	29 (14.7)
Carcinogenic substances, radiation, toxic chemicals, etc.		16 (3.2)	6 (1.9)	10 (5.1)
Hormones, Medicines, Contraceptives, etc.		24 (4.7)	22 (7.1)	2 (1)
Psychological factors		4 (0.8)	4 (1.3)	0 (0)
Obesity		12 (2.4)	12 (3.9)	0 (0)
Malnutrition		1 (0.2)	0 (0)	1 (0.5)
Smoking		1 (0.2)	1 (0.3)	0 (0)
I do not know		287 (56.7)	132 (42.7)	155 (78.7)
**Knowledge about breast cancer**				
Poor	Unacceptable level of knowledge	28 (5.5)	6 (1.9)	22 (11.2)
Low		196 (38.7)	97 (31.4)	99 (50.3)
Adequate	Acceptable level of knowledge	197 (38.9)	133 (43)	64 (32.5)
Excellent		85 (16.8)	73 (23.6)	12 (6.1)

Students' age > 20 years, studying at health colleges, studying in year 4 or above, having a higher monthly family income, and had a family member or relative with BC, had significantly higher mean knowledge scores compared with the other respective groups (*p* < 0.05). Mean differences in knowledge scores are presented in Table 5.

**Table 5:** Mean difference in BC knowledge score among participant groups (*N* = 506).

**Characteristics**	**Mean (SD)**	***P*-value**
**Age**		0.044
≤ 20 years	13.29 (3.83)	
> 20 years	14.19 (4.26)	
**College**		<0.001
Health colleges	15.20 (3.92)	
Non-health colleges	12.07 (3.85)	
**Marital Status**		0.348
Single	14.09 (4.15)	
Married	13.69 (4.26)	
**Study year**		0.027
3^rd^ year or less	13.45 (4.02)	
4^th^ years or above	14.31 (4.25)	
**Monthly family income**		0.005
SAR ≤ 10,000 (USD ≤ 2666.27)	13.49 (4.23)	
SAR > 10,000 (USD > 2666.27)	14.54 (4.05)	
**Any family member or relative had breast cancer?**		
Yes	14.68 (4.15)	0.049
No	13.79 (4.17)	

Regression analysis revealed that students' age, type of college type, and the presence of BC in family/relatives were significant predictors of BC knowledge. The knowledge score increases by 0.11 in students aged > 20 years (*p* < 0.05). In addition, the knowledge score decreases by 0.38 in students studying in non-health colleges (*p* < 0.001). In addition, the knowledge score of students without family/relatives affected by BC decreases by 0.119 (*p* < 0.01) (Table 6).

**Table 6:** Determinants of BC knowledge (*N* = 506).

**Variables**	**Model for knowledge of breast cancer**		
	**Coefficient (β) and (95% CI of β)**	***p*-value**	**VIF**
**Age** (≤ 20 years vs. > 20 years)	0.110 (0.048, 2.145)	0.040	1.72
**College** (Health vs. Non-health)	−0.380 (−3.96, −2.55)	<0.001	1.06
**Study year** (≤ 3^rd^ year vs. ≥ 4^th^ year)	0.008 (−0.83, 0.96)	0.885	1.71
**Monthly family income** (SAR ≤ 10,000, i.e., USD 2666.27) vs. SAR >10,000, i.e., USD 2666.27	0.053 (−0.24, 1.14)	0.204	1.06
**Any family member or relative had breast cancer?** (Yes vs. No)	−0.119 (−2.03, −0.39)	0.004	1.01

*Model fitness: ANOVA (F = 20.033, p <0.001), R^2^ = 0.17, adjusted R^2^ = 0.16*.

## Discussion

A novel aspect of this study was the inclusion of students from both health and non-health colleges. It is important to take into account because no comparative analysis has not reported in the available evidence (9, 12, 28). Moreover, this study used a previously validated questionnaire (2, 24). These aspects could be considered to be notable strengths of this study.

The majority of participants mentioned that their awareness regarding BC, mammography, screening, and treatment was not adequate, i.e., partially aware. More than half had heard about mammography. However, summing up the figures for partial and full awareness, it would increase to > 90% for BC awareness and screening and > 70% for mammography. A previous study reported that none of the female students studying health programs at this venue had undergone a mammography (9). In addition, our results were contradictory to the findings of Latif (9), with one-third of the students being aware of the treatment and slightly less than half of the health students being aware of the same.

In the study by Latif, 70.7% of the students scored high on knowledge of the diagnosis and treatment of BC (9). However, the study incorporated students from solely health disciplines. In our study, most participants (54.9%) were fully aware of BSE, while one-third were partially aware (34%) and more than half (51%) had performed BSE at home. This procedure was performed in similar proportions of health and non-health students, i.e., 54% and 46.2%. These findings were similar to those of a study conducted among Saudi nursing students in which 66% had BSE (28). However, our findings from health college students were much lower than the nursing student sample (28). A possible explanation to this occurrence is having students from other disciplines and not just nursing college. In another study, it was reported that 50.7% of female students studying health-related subjects at a public university in Dammam had BSE (9). Our health college data report a slightly higher proportion, i.e., 54%. Therefore, based on our findings, it is safe to state that the overall BC attribution of students has improved.

We found that a small proportion of students (8.1%) perceived media awareness as a possible reason for undergoing breast screening if necessary. This was similar to the findings of Alomair et al. as they mentioned media as an important source of information for students (12). However, more than a quarter of the health colleges sample and one-third of the non-health college sample perceived the fear of having BC as a probable reason to undergo screening if necessary. This finding was similar to the work of Naqvi et al. in Pakistani women (2). More than half of the participants at health and non-health colleges welcomed the notion of breast screening and agreed that it was better to undergo screening. In this context, female students at a public university in Riyadh also had positive attitudes toward screening techniques, such as BSE (12).

The majority of students mentioned that they are not embarrassed to talk about the issue in society and would prefer a female physician to discuss about BC. This was similar to the findings of Naqvi et al. (2). However, the majority of students in this study mentioned that they had never discussed BC with their doctor. This finding could be viewed from a different angle: in our study, as more than half of students were performing BSE at home. Hence, they may have felt empowered in self-management. In a previous study at this venue, a very small number of students (8.7%) underwent clinical breast examination while others (91.3%) did not (9). Hence, the number of students performing BSE has greatly increased, indicating better awareness of knowledge. It is important to mention here that available body of evidence does not demonstrate that regular BSE could lessen mortality attributable to BC. Though, it is crucial for patients to be aware of their normal breast size as well as vigilant enough to note and report any unusual changes to their practitioner (29).

Most students in our study (44.5%) mentioned that the main reason to undergo a screening would be to have an assurance of the absence of BC if necessary. A study in Pakistani women highlighted that 43.9% mentioned “ruling out the possibility of BC,” as a reason for the same (2). The majority of students (78.5%) advised that women must undergo breast screening which was, in a way, similar to the findings of Latif where most students correctly mentioned that early diagnosis improves treatment outcomes (9).

More than a quarter of students (26.5%) in this study mentioned healthcare professionals as a source of information regarding BC, followed by the internet and electronic media which 24.7% of students indicated. In this context, the health college sample was inclined towards healthcare professionals while non-health college students were inclined towards the internet and electronic media. Alomair et al. reported that most students (23%) highlighted media as the source of information while a small number of students (8.9%) mentioned health personnel as a source (12). This occurrence substantiates the importance of this medium in propagating knowledge contents about BC among the masses. In this context, the national health authorities have listed the month of October for the awareness of BC on their official website and routinely update its contents (30). Such an initiative has the potential to target the non-health student segment and improve their understanding of this disease.

Most students (53.6%) mentioned that BC was a major health issue in Saudi Arabia and rated it at seven on a scale of 10 in terms of its severity. This finding was similar to the response of Pakistani women (2). Barriers to screening reported in our study, such as culture/traditions that was reported by one-third of non-health college students, was also previously reported among adult women in Saudi Arabia (31). This highlights the existence of such barriers in the society and their continual inheritance among young people.

Half of the students (50.6%) in this study mentioned that obesity was not a risk factor for BC while slightly less than half (49.4%) mentioned otherwise. Breaking this sample by health and non-health colleges, more than half of students from health colleges mentioned obesity as a risk factor and a similar proportion from non-health colleges mentioned otherwise. In a previous study involving only students from health programs, the majority (62%) mentioned an incorrect response regarding the same (9). Most students in this study (56.7%) did not know the common symptom of BC while students in the study by Latif provided correct responses (9). However, students in our study identified family history as a risk factor for BC. This finding was similar to the results of a previous study at the same venue (9). To summarize these findings, the sample in our study had a better understanding of the urgency of the problem and its risk factors. Though, more efforts are needed in this direction.

Nearly 40% of the sample had adequate knowledge while nearly 17% had excellent knowledge. Taking these figures together, approximately 55% of students had an acceptable level of knowledge. Distributing the responses based on health and non-health colleges, 43% of the students from health colleges had adequate knowledge while nearly a quarter had excellent knowledge (23.6%). Taking these figures together, nearly 70% of health college students had an acceptable level of knowledge. On the other hand, just over one-third of students in non-health colleges had adequate knowledge (32.5%) while 6.1% had excellent knowledge. Combining these figures would result in almost 39% of non-health college students having an acceptable level of knowledge. Because the findings from a study by Latif on health-subject students showed that 70% of the respondents scored ≥ 50%, our findings are similar, if not better (9). In addition, a study at a university in Riyadh, which included both health and non-health program students highlighted a moderate level of knowledge (12). In the sample of Pakistani women including students, the majority had low knowledge (2).

Furthermore, statistical analysis highlighted that there was a mean difference in knowledge scores, and knowledge scores decreased by 0.38 for students studying at non-health colleges (*p* < 0.001). Students studying in health colleges had slightly higher scores compared to students studying at non-health colleges This aspect has never been investigated before in any of the previous studies (9, 12, 24, 28). An explanation for this occurrence could be the content of health education. Students studying in health programs may have some educational modules in their curriculum. This notion is supported by a study on Saudi nursing students at a university in Riyadh where more than half (62%) of participants mentioned that they learned BSE in college curricula. Moreover, there was a positive correlation between the practice of BSE and academic experience (28). This highlights the impact of health education on the knowledge and daily practices of students pertaining to breast screening.

While it is understood that students from non-health colleges may not have such health education embedded in the curriculum, it is worthwhile mentioning that the implementation of BC awareness campaigns at the university level will definitely help empower this student population. Hence, to reiterate the point made earlier, such campaigns have been constantly organized every year by the university where health experts provide education about this disease.

In addition, statistical analysis revealed that students with family/relatives suffering from BC tend to have higher knowledge as compared to those who did not. The knowledge score of students with no family/relatives suffering from BC decreased by 0.119. This could be explained by having previous experience with the disease. Encountering a patient with BC may provide an experience that may prompt a caregiver to seek further knowledge regarding the disease. This knowledge enhancement depends on the nature and extent of the encounter. A study to support this notion would be the work of Naqvi et al. where cross-tabulation of the variable of knowledge with, “BC in the family” and “anyone outside of family having BC” showed a significant association (*p* < 0.01) and participants who had an interaction with someone having BC, showed better knowledge (2).

The sampling strategy used in the study is convenience sampling, which unfortunately does not provide a representative sample. However, this strategy has the advantage of helping to gather data from a large number of participants from health and non-health colleges, particularly in COVID-19. Therefore, the findings from this study must be interpreted considering this aspect.

## Conclusion

The findings of the surveyed students suggested that more than half of the students had an acceptable level of knowledge. By education sector, approximately 70% and 40% of students from health and non-health colleges, respectively, had an acceptable level of knowledge. By comparing it with previous evidence, the knowledge of BC has significantly improved. The role of awareness campaigns as an information medium for students from non-health backgrounds is greatly appreciated. Moreover, the internet and electronic media have emerged as new sources of information for non-health students, and therefore, more efforts are needed to utilize this medium in empowering this student population in understanding of this disease.

There is no available evidence that reports the knowledge of non-health students; therefore, these findings from this study can serve as a primary data source for the same. The disease was perceived in a similar way regardless of health education. This aspect may prove helpful in mass disease education initiatives as the society is already prepared mentally to acknowledge, understand, and mitigate the risk of BC. The study would provide the evidence to support the design of targeted campaigns aimed at improving knowledge. This will set a good precedent for others to carry out such studies in their respective educational institutes as other educational institutes of the country have also started such campaigns (32–34).

## Data Availability Statement

The original contributions presented in the study are included in the article/supplementary materials, further inquiries can be directed to the corresponding author.

## Ethics Statement

The studies involving human participants were reviewed and approved by Institutional Review Board of King Abdullah International Medical Research Center (Study# RA20/020/A) and (Reference# IRBC/1325/20). The patients/participants provided their written informed consent to participate in this study.

## Author Contributions

All authors listed have made a substantial, direct, and intellectual contribution to the work and approved it for publication.

## Conflict of Interest

The authors declare that the research was conducted in the absence of any commercial or financial relationships that could be construed as a potential conflict of interest.

## Publisher's Note

All claims expressed in this article are solely those of the authors and do not necessarily represent those of their affiliated organizations, or those of the publisher, the editors and the reviewers. Any product that may be evaluated in this article, or claim that may be made by its manufacturer, is not guaranteed or endorsed by the publisher.
